# The Voluntary Hospitals and Grants in Aid

**Published:** 1914-06-27

**Authors:** 


### The Hospital

**The Workers' Newspaper of Administrative Medicine and Institutional Life, Administration, National Insurance and Health.**

No. 1462, Vol. LVI. SATURDAY, JUNE 27, 1914. PRICE ONE PENNY.

### THE VOLUNTARY HOSPITALS AND GRANTS IN AID.

The first day's proceedings at the meeting of the British Hospitals Association at Newcastle-on-Tyne included a paper by Dr. Hume on "The Voluntary Hospital on its Trial." (See p. 349 *et seq.*) The discussion which this paper called forth, which included a speech by Mr. Straker, corresponding secretary of the Northumberland Miners' Association, cannot fail to interest hospital managers throughout the country. Mr. Straker is a declared and advanced Socialist. He wants State hospitals, of the consequences of which (as became apparent to those present) he has little or no knowledge; he is most anxious that the circumstances of the working man, and indeed of all applicants for hospital relief, should not be inquired into; and he favours, as an alternative, the opening of pay-wards in connection with all the large hospitals, to which those members of the working classes who so desire can obtain admission by payment. The Newcastle meeting brought out and confirmed a view which has been presented to us from various parts of the country. This is that the working classes desire individually and in large numbers not to accept free medical relief, whether it be provided by the benevolent or by the municipalities out of the rates. Their view seems to be that every man should have the right to pay what he can towards the cost of his maintenance and treatment in hospital, and it is encouraging to find that recent legislation, including the Workmen's Compensation Acts and National Insurance, should have so improved the resources of the working classes as to make it possible for them to take up this attitude in regard to hospital treatment.

The discussion on Dr. Hume's paper soon revealed that neither Dr. Hume nor Mr. Straker, who advocated State hospitals, was familiar with the present position of hospital provision in Great Britain. They were evidently unaware that the voluntary hospitals, despite the heavy additional burden cast upon them by the provision of in-patient treatment for insured persons, have weathered the storm and proved that they can carry on the work with increased efficiency, providing, as we have always contended, that the Insurance Acts are amended or that power is given to the municipalities throughout the country, subject to the sanction of the Treasury or the Local Government Board, to make grants in aid as occasion may arise, with the object of enabling the voluntary hospitals to make provision for and treat as in-patients such insured persons as the Commissioners may find it necessary to provide for. Dr. Hume and Mr. Straker failed to grasp the point that there is no call for State hospitals because there is no need for the State provision of hospitals, as the present position shows, and they seem to have no apprehension of grants in aid which have been recognised by Parliament already in connection with voluntary hospitals, notably in the case of Liverpool.

The position of the voluntary hospitals at the present time is this. They treated some 50,000 insured persons as in-patients during 1913. Of this number a percentage consisted of patients who would have received in-patient treatment in the ordinary course had there been no National Insurance. The remainder represented patients which have not heretofore found their way to the hospitals, and who have now presented themselves owing mainly to the working of National Insurance through the panel system. This new army of hospital recruits has not only filled to repletion all the available beds in most of the principal hospitals throughout the country, but it has entailed large additions to the waiting-lists of cases urgently needing in-patient treatment. So far as we can at present estimate the probable numbers involved in the hospitals of Great Britain, we believe it would be found that an extra 10,000 or at the outside 15,000 hospital beds would represent the extra accommodation demanded by insured persons. Many hospital beds can readily be provided by making available existing hospital buildings scattered up and down the country, and in the Metropolis through Poor-Law infirmaries and Union Poor-Law hospitals. These buildings are far too costly and up to date to be maintained for the reception of chronics, imbeciles, dements, and other waste products of our civilisation. We have carefully inspected a great number of these buildings, and we find that they could be readily made into up-to-date hospitals by the expenditure of relatively small sums, and that by increasing the medical staff, and where necessary adding an operation unit, and by the appointment of a visiting surgeon or surgeons and a physician they might be turned at once into State hospitals free from any taint of pauperism.

The position of the voluntary hospitals financially is sound and remarkable. Thus the income of the voluntary hospitals has increased in seventeen years by 69 per cent.—that is from about £2,500,000 in 1896 to upwards of £4,500,000 in 1912. Naturally, the ever-increasing demands of scientific treatment have raised the annual expenditure of all voluntary hospitals; but on the whole ends have met, and we find the average surplus income of the voluntary hospitals collectively during the four years ending 1912 has amounted to upwards of £330,000 per annum. A full consideration of the actual cost entailed on the voluntary hospitals by the National Insurance Acts demonstrates that, if the Treasury would sanction an annual grant in aid on behalf of the in-patient treatment given to the insured, of from £250,000 to £500,000, the voluntary hospitals of this country could successfully carry on all the work entailed upon them through the Insurance Acts under existing circumstances, and at the same time meet all their liabilities with reasonable facility. In these days a maximum of £500,000 is a sum of relatively small importance to a Chancellor of the Exchequer. The figures we have quoted demonstrate further that the voluntary hospital system is sound and safe, for the requirements of the population do not represent an annual expenditure of £20,000,000, the cost of State hospitals, but grants in aid amounting in the whole, we estimate, ultimately to two millions a year, of which one-fourth to one-half would go to voluntary hospitals, and the remaining half to the reorganised and remodelled State hospitals placed under municipal management, and created in the way we have indicated already. The whole country is in favour of the course here suggested, and the sooner the Government call in expert assistance and get together all the facts the better claim will its members have to the title of statesmen.

